# Meshless Method with Operator Splitting Technique for Transient Nonlinear Bioheat Transfer in Two-Dimensional Skin Tissues

**DOI:** 10.3390/ijms16012001

**Published:** 2015-01-16

**Authors:** Ze-Wei Zhang, Hui Wang, Qing-Hua Qin

**Affiliations:** 1 Research School of Engineering, Australian National University, Acton, ACT 2601, Australia; E-Mails: zewei.zhang@anu.edu.au (Z.-W.Z.); qinghua.qin@anu.edu.au (Q.-H.Q.); 2 Institute of Scientific and Engineering Computation, Henan University of Technology, Zhengzhou 450001, China

**Keywords:** transient nonlinear bioheat transfer, meshless method, operator splitting, radial basis function, method of fundamental solutions

## Abstract

A meshless numerical scheme combining the operator splitting method (OSM), the radial basis function (RBF) interpolation, and the method of fundamental solutions (MFS) is developed for solving transient nonlinear bioheat problems in two-dimensional (2D) skin tissues. In the numerical scheme, the nonlinearity caused by linear and exponential relationships of temperature-dependent blood perfusion rate (TDBPR) is taken into consideration. In the analysis, the OSM is used first to separate the Laplacian operator and the nonlinear source term, and then the second-order time-stepping schemes are employed for approximating two splitting operators to convert the original governing equation into a linear nonhomogeneous Helmholtz-type governing equation (NHGE) at each time step. Subsequently, the RBF interpolation and the MFS involving the fundamental solution of the Laplace equation are respectively employed to obtain approximated particular and homogeneous solutions of the nonhomogeneous Helmholtz-type governing equation. Finally, the full fields consisting of the particular and homogeneous solutions are enforced to fit the NHGE at interpolation points and the boundary conditions at boundary collocations for determining unknowns at each time step. The proposed method is verified by comparison of other methods. Furthermore, the sensitivity of the coefficients in the cases of a linear and an exponential relationship of TDBPR is investigated to reveal their bioheat effect on the skin tissue.

## 1. Introduction

It is widely recognized that accurate and fast prediction of temperature distribution in biological tissues is important in various practical diagnostics. For example, doctors would like to know the heat and temperature changes during surgery on a skin tumor or the human eye so that they can adjust the power of the laser therapy to avoid extra burning injury of healthy tissue [1,2]. Due to the complexity of the bioheat system itself, numerical methods have been overwhelmingly utilized for noninvasive diagnostics. Several linear or nonlinear steady-state bioheat models involving changed thermal conductivity and blood perfusion rate have been numerically solved to analyze the induced temperature distribution in biological tissues [3,4,5,6,7,8]. Besides, a non-Fourier heat conduction model in one-dimensional multilayered systems was analyzed by Laplace transform and the fast inversion technique [9,10,11]. In this paper, a model for describing transient nonlinear bioheat transfer model in two-dimensional (2D) skin tissue is developed.

In the context of transient nonlinear bioheat transfer, when there is a need to dynamically monitor the changes of temperature in time and space during the bioheat transfer process, some numerical models have been developed for various biological tissues. For instance, Arunn *et al.* [12] investigated the variation of transient temperature of a 2D human eye computational model using the finite volume method. Trakic *et al.* [13] predicted the transient temperature rise in a nonlinear heat transfer model of tumor and healthy tissue of mouse by the commercial finite element software FEMLAB. Feng *et al*. [14] applied finite element technology coupled with a nested-blocked optimization algorithm to predict the temperature distribution in a prostate during a nanoshell-mediated laser surgery.

Boundary-type methods, which are different from the domain-type methods above, have also been presented to solve such problems. Among them, the dual reciprocity boundary element formulation has been proposed for analysis of the transient nonlinear 2D bioheat transfer system subjected to a sinusoidal heat flux on the skin surface [15]. An axisymmetric boundary element formulation using the time-dependent fundamental solution was derived by Majchrzak for the analysis of freezing and thawing processes in biological tissues [16]. Alternatively, Cao *et al.* [17] developed a mixed meshless method RBF-MFS by coupling the radial basis function (RBF) and the method of fundamental solutions (MFS) to analyze the transient linear thermal behavior in skin tumor tissue. It is noted that in these numerical methods, the Laplace transform method or finite difference technology with respect to time were applied to handle the time variable in the bioheat transfer governing equation. However, the Laplace transform method is usually limited to linear transient problems [18]. For other cases, the finite difference scheme needs carefully consideration of the time step length to obtain accurate, stable, and convergent results [19]. These difficulties have motivated researchers to develop other methods for effectively handling the time derivative term and for approximating the nonlinear source terms in the governing equation. For example, the operator splitting method for uniformly handling the transient term and nonlinear source term explicitly using two-level higher-order time step schemes has received considerable attention [20,21].

In this work, we aim to develop a mixed meshless method for analyzing transient nonlinear bioheat transfer in 2D skin tissue by way of the operator splitting method. In the proposed solution procedure, the operator splitting method is employed first to isolate the transient and nonlinear terms in the original Penney bioheat governing equation by the explicit second-order Adams-Bashforth time marching for the half time step and the second-order Adams-Moulton scheme for the next time step. Then, the new equation in the form of a modified Helmholtz equation can be derived and solved at each time step. Next, the mesh-free dual reciprocity method implemented by the RBF interpolation and the mesh-free MFS in terms of fundamental solution kernels are respectively utilized to determine the particular solutions and the homogeneous solutions of the modified Helmholtz problem at each time step by simple internal and boundary collocations. The advantages of this meshless scheme are that only the boundary integrals are included in the calculation process. Thus the computational time is less than the corresponding finite element method. Secondly there will be no singular integrals generated in the process, because the source points and field points are placed outside the solution domain respectively. There is no complicated and time-consuming mesh generation required during the process. However, the disadvantage of the proposed meshless scheme is that it is difficult to deal with multi-material problems, compared to the conventional finite element method based on feasible element material definition.

The paper is organized as follows: In Section 2, the numerical results from the proposed numerical method are verified by comparison with those from ANSYS software. Sensitivity analysis of the effect of coefficients on temperature distribution in the case of temperature-dependent blood perfusion rate is performed in the same section. In Section 3, the mathematical bioheat model of 2D skin tissue is described and in Section 4, the solution procedure including the operator splitting method, the dual reciprocity method, and the MFS is presented. Finally, some conclusions are drawn in Section 5.

## 2. Numerical Results and Discussion

### 2.1. Verification of the Proposed Method

In this section, the efficiency and accuracy of the proposed method for analyzing transient nonlinear bioheat transfer in 2D skin tissue are validated by the finite element software ANSYS through a benchmark example. In this work, we consider the nonlinear term induced by the blood perfusion rate, which is a function of the tissue temperature. The thermal parameters of the 2D skin tissue model used in the calculation are given in Table 1 [17,22,23].

**Table 1:** Thermal properties of the skin.

Thermal Parameters	Value
Thermal conductivity *k*	0.5 Wm^−1^·K^−1^
Density of blood *ρ_b_*	1000 kg·m^−3^
Specific heat of blood *c_b_*	4200 J·kg^−1^·K^−1^
Spatial heat *Q_r_*	30,000 Wm^−3^
Metabolic heat *Q_m_*	4200 Wm^−3^
Arterial temperature *T_b_*	37 °C
Temperature of body core *T_c_*	37 °C
Temperature of skin surface *T_s_*	25 °C

**Figure 1:**
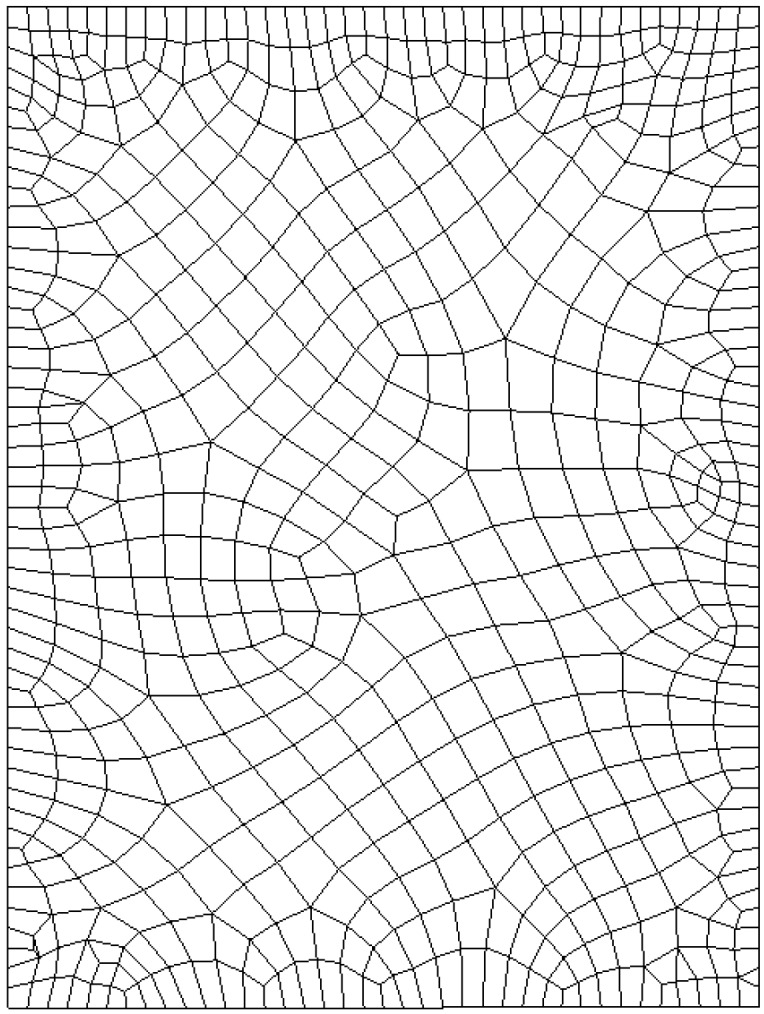
Finite element mesh used in ANSYS.

The ANSYS transient thermal toolbox is employed to simulate bioheat transfer in the biological material. The mesh generated by ANSYS is shown in Figure 1, in which 647 elements and 733 nodes are generated for finite element analysis.

**Figure 2:**
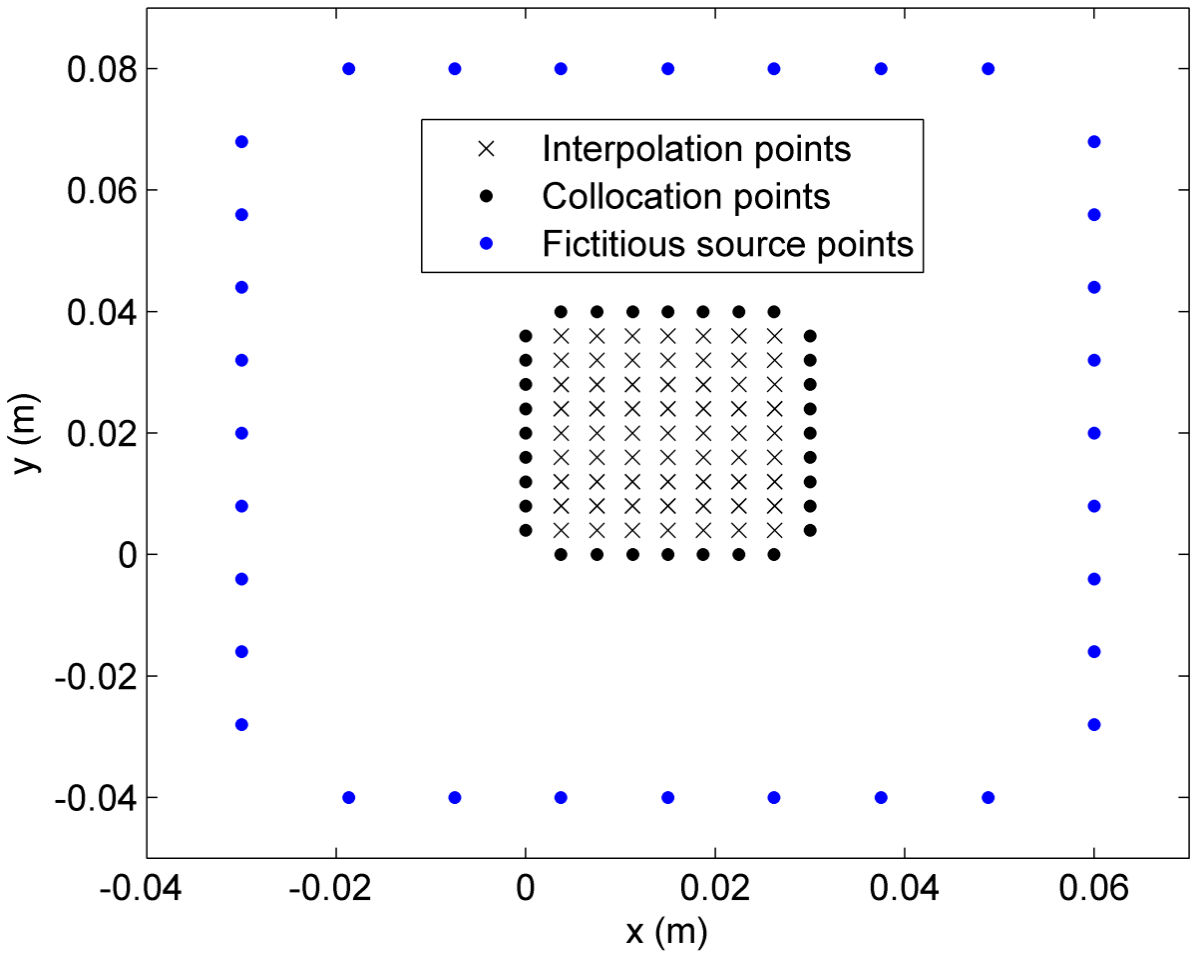
Collocation scheme with 63 interpolation points and 32 boundary collocations.

**Figure 3:**
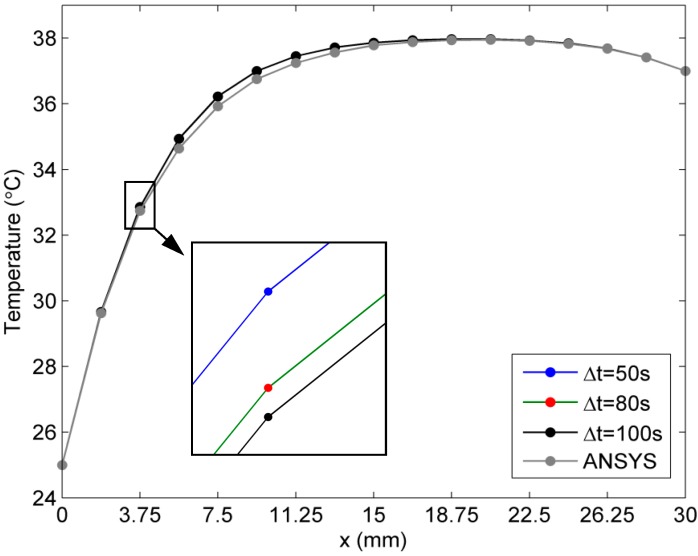
Results for the linear case of blood perfusion rate (The arrow is indicator of zoom in image of the three overlap points in temperature curves. So that the reader can view the curves in details clearly).

For the purpose of comparison, we consider that blood perfusion rate is a linear function of tissue temperature: $\omega_{b}(T)=a_{1}+a_{2}T$, where $a_{1}=0.0005$ and $a_{2}=0.0001$. 63 interpolation points and 32 boundary collocations (see Figure 2) are used to calculate the transient temperature distribution. Numerical results along the *x*-axis at three time instants *∆t* = 50, 80, and 100 s are presented in Figure 3 to show the accuracy and stability of the second-order Adams-Bashforth and Adams-Moulton schemes. From Figure 3, it can be seen that the results from the proposed algorithm with fewer collocation points are in good agreement with the results from the ANSYS Transient thermal toolbox. The relative error of the results from the proposed method with respect to those from the Transient thermal toolbox of ANSYS is less than 0.5%.

**Figure 4:**
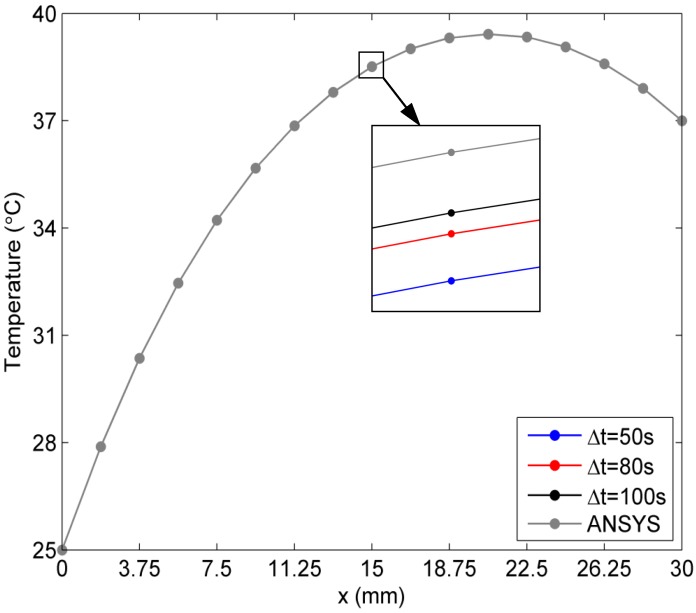
Results for the exponential case of blood perfusion rate (The arrow is indicator of zoom in image of the four overlap points in the temperature curves. So that the reader can view the curves in details clearly).

Next, the exponential case of the temperature-dependent blood perfusion rate $\omega_{b}(T)=a_{1}e^{a_{2}T}$ with $a_{1}=0.0005$ and $a_{2}=0.01$ is considered. Again, numerical results along the *x*-axis at three time instants *∆t* =50 s, 80 s, and 100 s are evaluated and shown in Figure 4. It is evident that there is negligible difference between the results from the proposed algorithm and those from the ANSYS Transient thermal toolbox.

**Figure 5:**
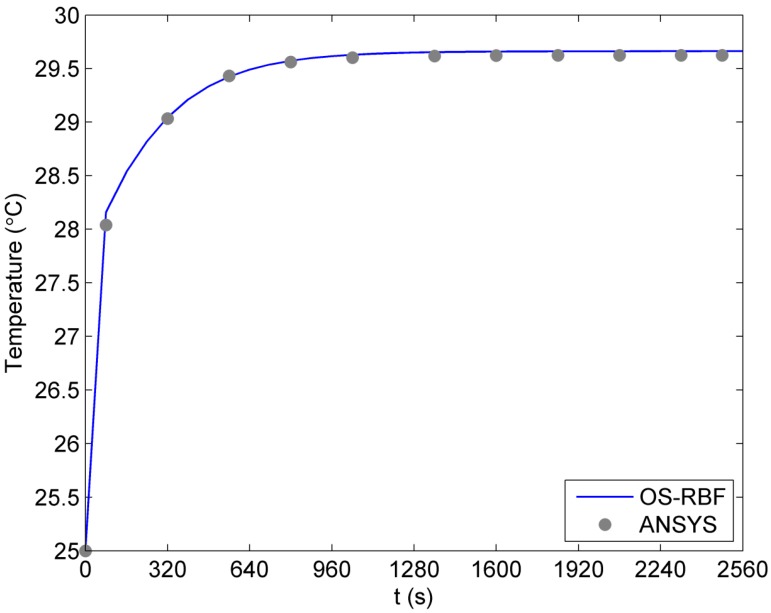
Variation of temperature with time for the linear case of blood perfusion rate.

Further, Figure 5 presents the temperature variation from *t* = 0 s to *t* = 2560 s at the point (1.875 mm, 0) on the *x*-axis for the case of a linear blood perfusion rate. It can be seen from Figure 5 that the variation of temperature with time from the proposed meshless method is almost identical to that obtained from ANSYS, although much less unknowns are used in the proposed method.

Thus, the convergence and accuracy of the present meshless method with the higher-order AB and AM time-stepping schemes is validated for transient nonlinear bioheat analysis in the rectangular model of skin tissue.

**Figure 6:**
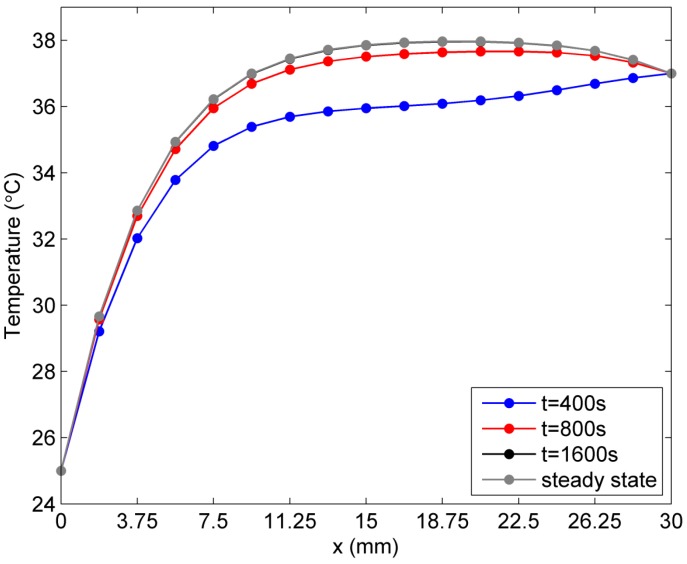
Temperature variation *vs.* time along *x*-axis for the linear-form blood perfusion rate.

**Figure 7:**
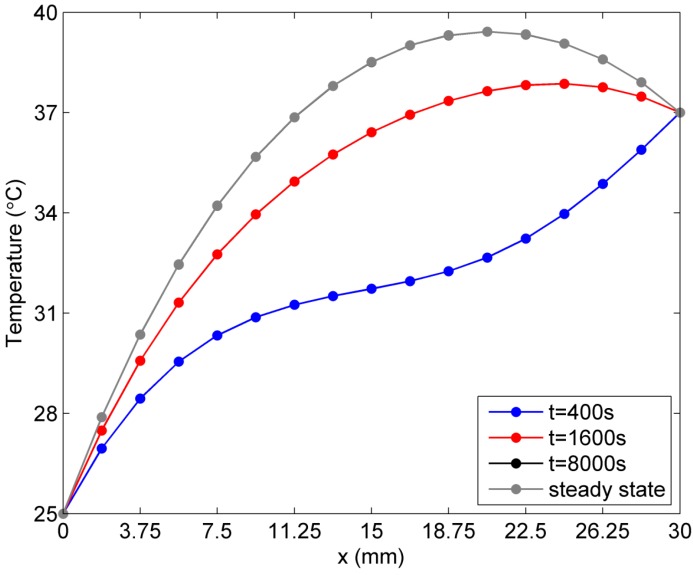
Temperature variation *vs.* time along *x*-axis for the exponent-form blood perfusion rate.

More numerical results are now presented to illustrate temperature distribution in the solution domain caused by different temperature-dependent blood perfusion rates. In Figure 6 and Figure 7, the temperature distribution in the skin tissue along the *x*-axis at different times is presented. It is found that the steady state in Figure 6 is reached much earlier (the linear case, at about 1600 s) than that in Figure 7 (the exponential case, at about 8000 s). It is also noted that the slope of the steady-state temperature curve along the *x*-axis increases and then decreases from the left side to the right side for both linear and exponential cases. However, the slope for the linear case appears greater than that for the exponential case in the region close to the left surface, which has a lower environmental temperature, whereas the slope for the linear case becomes less than that for the exponential case in the region close to the right surface, which has a higher body core temperature. Moreover, the exponential-form blood perfusion rate produces a higher interior temperature in the region close to *x* = 18.75 mm than that for the linear-form rate. The main reason is that the exponential-form blood perfusion rate generally has a lower value of the blood perfusion rate than the linear-form with the coefficients given above. In the region close to the left surface, where the skin tissue temperature is evidently lower than the blood temperature, the greater blood perfusion rate means that more heat flows from blood to skin tissue, causing a rapid increase of the tissue temperature. Thus there is greater temperature gradient in this region for the linear case than the exponential case. When the tissue temperature exceeds the blood temperature, a greater blood perfusion rate causes more heat to flow from tissue to blood and causes the tissue temperature to decrease.

### 2.2. Sensitivity of Temperature to Variation of Constants a_i_ in Linear Case of Blood Perfusion Rate

**Figure 8:**
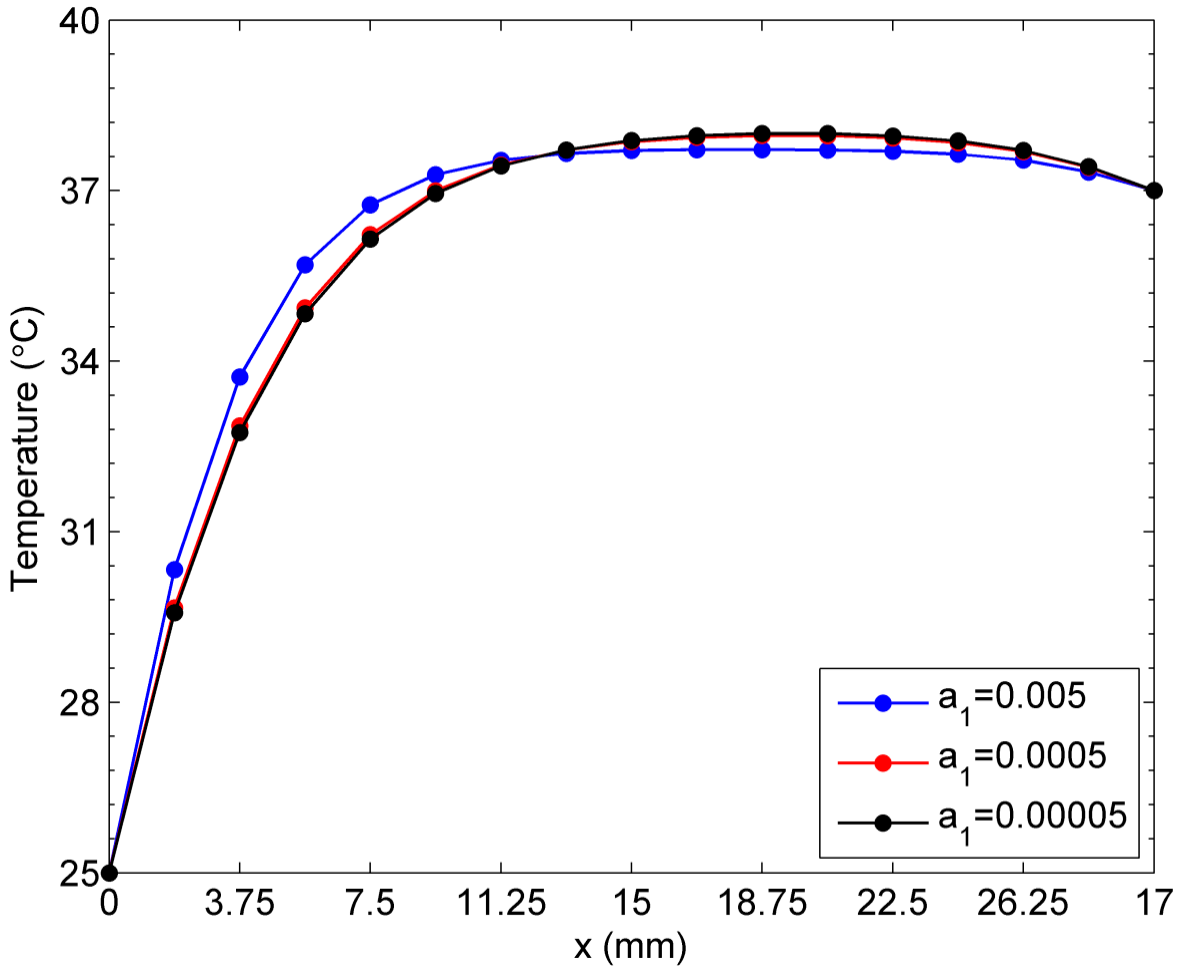
Sensitivity of temperature to constant *a*_1_ in the linear case of blood perfusion rate.

In this section, the linear case of temperature-dependent blood perfusion rate $\omega_{b}(T)=a_{1}+a_{2}T$ is considered for the sensitivity analysis of temperature to the constant coefficients $a_{1}$ and $a_{2}$. Firstly, the coefficient *a_2_* is set to be constant 0.0002 and the constant $a_{1}$ is assumed to be 0.005, 0.0005, and 0.00005, respectively. As we can see in Figure 8, the steady-state temperatures are quite close to each other when $a_{1}=0.00005$ and $a_{1}=0.0005$. But the skin temperature curve has a relative larger gap with the two curves mentioned above when $a_{1}=0.005$. In addition, the three temperature curves intersect at the point (12.65 mm, 0), at which the skin temperature is approximately 37 °C. Therefore, from the left surface of the skin tissue to the approximate location point (12.65 mm, 0), the greater blood perfusion rate indicates that more heat flow transfer occurs between the blood and skin tissue. If the blood temperature is higher than the skin tissue, more heat flow transferred from blood to skin tissue causes a rapid increase in skin temperature. If the blood temperature is lower than the skin tissue, more heat flow transferred from skin tissue to blood causes a rapid decrease in skin temperature. It can be seen that blood perfusion protects the skin tissue from extreme temperature increases or decreases caused by the environment.

**Figure 9:**
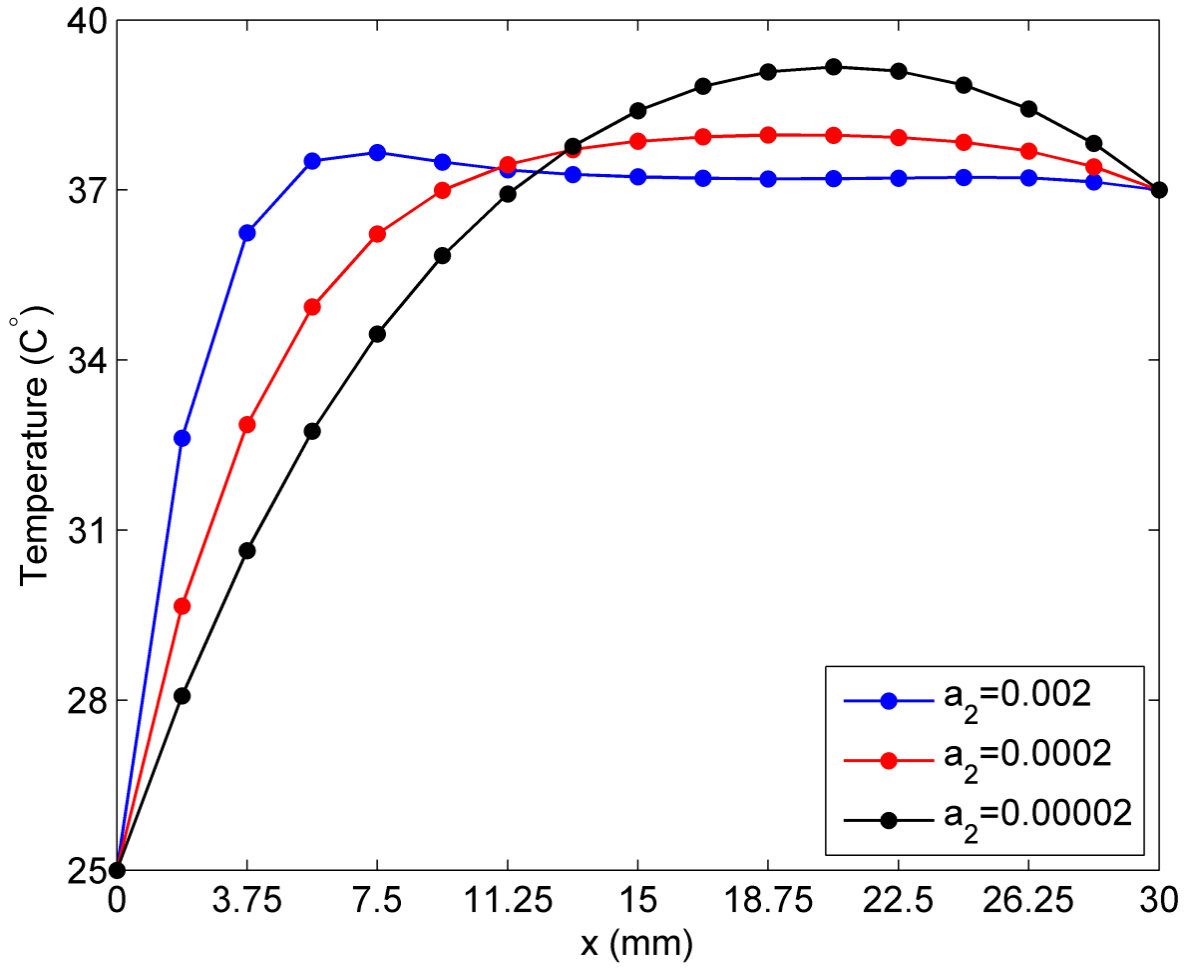
Sensitivity of temperature to constant *a*_2_ in the linear case of blood perfusion rate.

To study the effect of *a*_2_ on skin temperature, we assume the first constant $a_{1}$ to be 0.0005 and the second constant $a_{2}$ is set as 0.00002, 0.0002, and 0.002. From Figure 9, it can be seen that variation of the constant $a_{2}$ causes a more rapid change in the steady-state temperature curve than that due to variation of the constant $a_{1}$. In particular, when the constant $a_{2}$ = 0.002, the curve of the tissue temperature is steeper than the other two curves. The highest value of the skin temperature appears at the approximate location (7.5 mm, 0), which is closer to the left hand side boundary of the skin tissue than the other two curves. From the location (15 mm, 0) to (26.25 mm, 0), the skin tissue temperature is stable at a certain level when the constant $a_{2}$ = 0.002.

### 2.3. Sensitivity of Temperature to Variation of a_i_ in the Exponential Case of Blood Perfusion Rate

**Figure 10:**
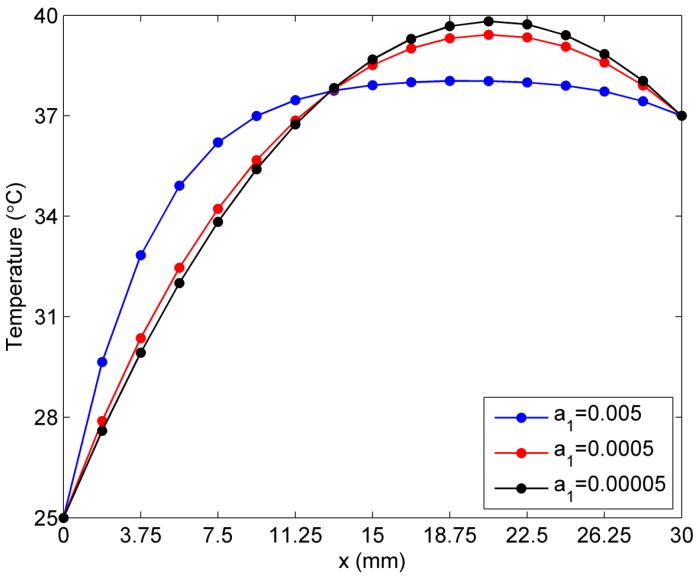
Sensitivity of temperature to constant *a*_1_ in the exponential case of blood perfusion rate.

In this section, the sensitivity analysis of temperature to the constant coefficients $a_{i}$ is investigated by considering the exponential case of temperature-dependent blood perfusion rate $\omega_{b}(T)=a_{1}e^{a_{2}T}$. When the constant $a_{2}$ is assumed to be 0.01, the constant $a_{1}$ is respectively tested at 0.005, 0.0005, and 0.00005. Compared with the linear case shown in Figure 8, the difference or gap between each skin temperature curve is relative larger, as shown in Figure 10. Similarly, the three temperature curves with different values of constant $a_{1}$ intersect at almost the same point (the distance from the left hand side boundary being roughly 13.125 mm). This finding means that, at the location (13.125 mm, 0), the skin temperature has almost the same value of 37.75 °C for different values of the constant $a_{1}$. Figure 10 illustrates the stronger regulatory and protective effect of the exponential-form blood perfusion rate than that in the linear case (see Figure 8).

**Figure 11:**
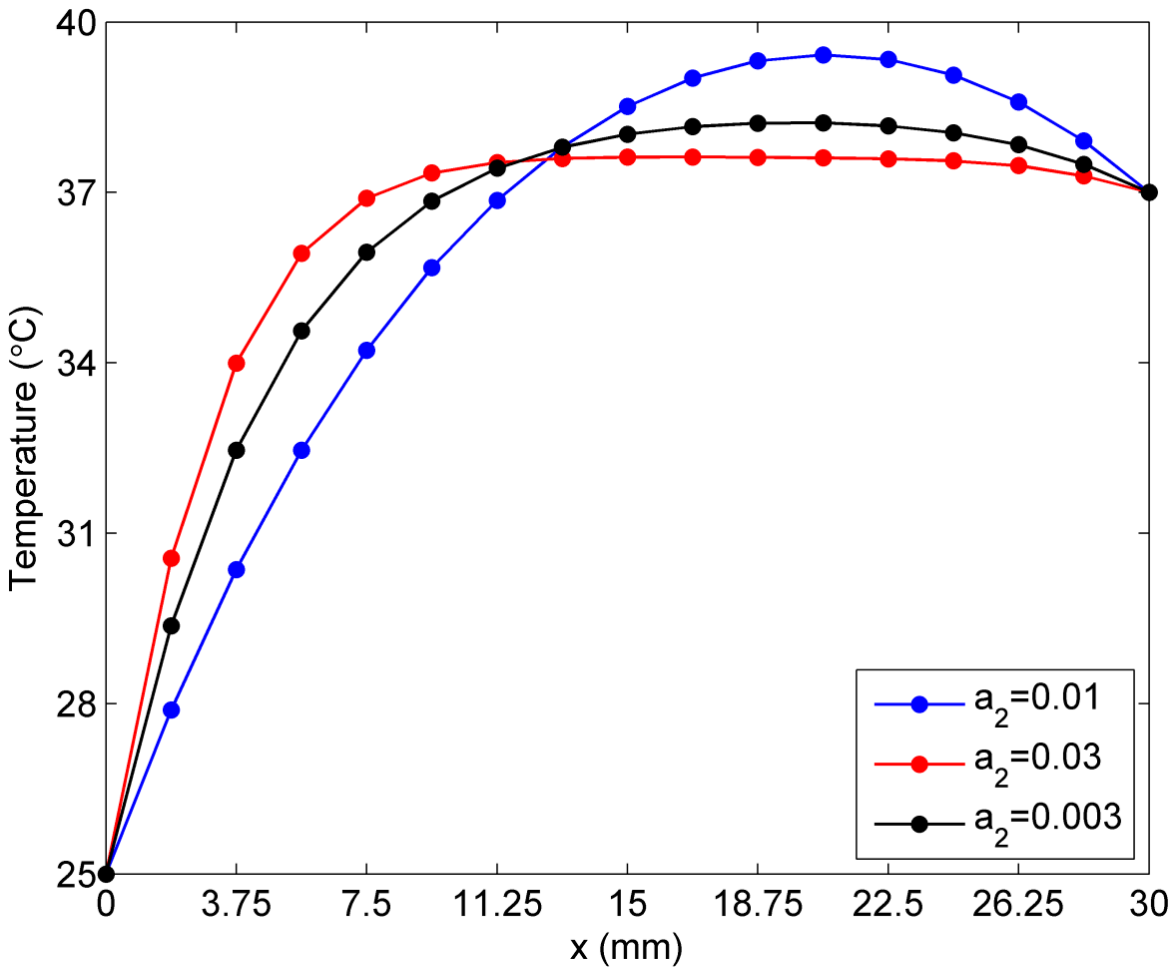
Sensitivity to constant *a*_2_ in the exponential case of blood perfusion rate.

Again, we assume constant *a*_1_ to be 0.0005, while constant $a_{2}$ is set to be 0.03, 0.01, and 0.003. As we can see from Figure 11, when constant $a_{2}$ = 0.03, the temperature of the skin tissue becomes increasingly steep before the point (11.25 mm, 0), but the curve is flatter than temperature curves with smaller values of $a_{2}$. Compared with the effect of the different values of $a_{1}$ in Figure 10, the increase in the value of $a_{2}$ causes a larger reduction of the peak value of the skin tissue temperature and the temperature becomes more stable from the location (11.25 mm, 0) to (26.25 mm, 0). In summary, an increase in the value of constant $a_{2}$ has a higher sensitivity to the temperature of skin tissue than an increase in the value of constant $a_{1}$. Simultaneously, it is found that an increase in the blood perfusion rate causes the temperature of the skin tissue to reach its final steady state more quickly and reduces the peak value of the tissue temperature. That means that if the skin tissue absorbs a large quantity ofbiological heat from its environment, the blood perfusion effect causes the temperature to reach a certain value quickly and reduces the risk of burning of the skin tissue.

## 3. Mathematical Bioheat Transfer Model in 2D Skin Tissue

The transient heat transfer in biological tissue is usually governed by the well-known Pennes’s bioheat transfer equation [7,24]:

(1) $$k\nabla^{2}T(\text{x},t)+\rho_{b}c_{b}\omega_{b}\left(T_{b}-T(\text{x},t)\right)+Q_{r}+Q_{m}=\rho c\frac{\partial T(\text{x},t)}{\partial t}$$

where *T* is the temperature, ρ the tissue density, *c* the tissue specific heat, *k* the tissue thermal conductivity, $\rho_{b}$ the blood density, $c_{b}$ the blood specific heat, $\omega_{b}$ the blood perfusion rate, $T_{b}$ the arterial temperature, $Q_{r}$ the spatial heat sources, $Q_{m}$ the metabolic heat generation rate, *t* the time, and $\nabla^{2}$ the standard Laplacian operator.

In practice, blood flow accelerates with the increase in temperature of the environment. Thus, blood perfusion rate can be viewed as a function of tissue temperature. In this case, the governing Equation (1) can be written in the form of nonlinear equation as follows:

(2) $$k\nabla^{2}T(\text{x},t)+\rho_{b}c_{b}\omega_{b}(T)\left(T_{b}-T(\text{x},t)\right)+Q_{r}+Q_{m}=\rho c\frac{\partial T(\text{x},t)}{\partial t}$$

In hyperthermia treatment, the blood perfusion is usually assumed to vary linearly [15,22,25]:

(3) $$\omega_{b}\left(T\right)=a_{1}+a_{2}T$$

or exponentially [5,22,26]:

(4) $$\omega_{b}\left(T\right)=a_{1}e^{a_{2}T}$$

with the tissue temperature *T*. $a_{1}$ and $a_{2}$ are positive constants.

For the sake of convenience, we introduce a new temperature variable θ:

(5) $$\theta =T-T_{b}$$

then, the nonlinear governing Equation (2) can be rewritten in terms of the new variable as:

(6) $$k\nabla^{2}\theta -\rho_{b}c_{b}\omega_{b}(\theta +T_{b})\theta +Q_{r}+Q_{m}=\rho c\frac{\partial \theta }{\partial t}$$

or

(7) $$\frac{k}{\rho c}\nabla^{2}\theta -\frac{\rho_{b}c_{b}}{\rho c}\omega_{b}\left(\theta +T_{b}\right)\theta +\frac{Q_{t}}{\rho c}=\frac{\partial \theta }{\partial t}$$

where

(8) $$Q_{t}=Q_{r}+Q_{m}$$

represents the generalized interior heat source term including the metabolic heat of the tissue and the spatial heat source caused by laser heating.

Further, Equation (7) can be expressed in the general unsteady Poisson equation form as:

(9) $$\frac{\partial \theta }{\partial t}=\frac{k}{\rho c}\nabla^{2}\theta +f\left(\theta \right)$$

with

(10) $$f\left(\theta \right)=-\frac{\rho_{b}c_{b}}{\rho c}\omega_{b}\left(\theta +T_{b}\right)\theta +\frac{Q_{t}}{\rho c}$$

**Figure 12:**
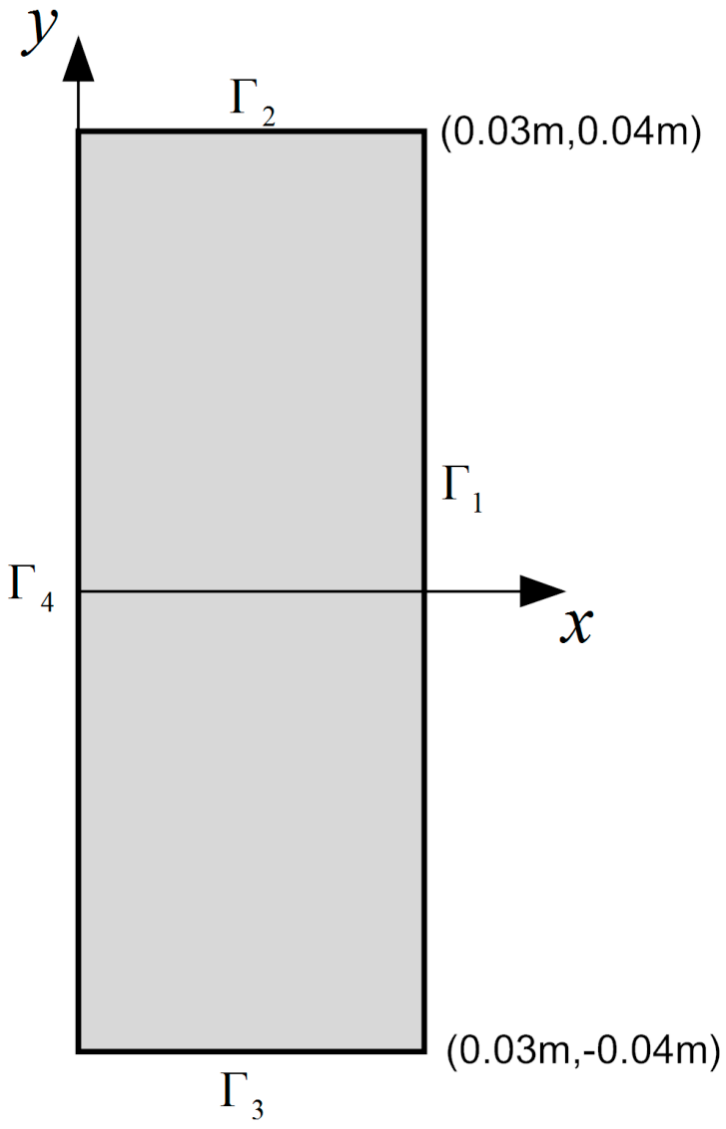
Two-dimensional skin model.

Besides the governing Equation (9), boundary conditions of the problem that describes bioheat transfer in a rectangular skin domain as shown in Figure 12 include [4,15,27]:

(1) the temperature condition at the right boundary $\Gamma_{1}$, that is assumed to be the body core temperature $\theta_{c}$:

(11) $$\theta =\theta_{c}\;\;\;\;\;\;\;\;\;\;\text{at}\;\text{boundary}\;\Gamma_{1}$$

(2) the thermal insulation conditions at the upper boundary $\Gamma_{2}$ and the lower boundary $\Gamma_{3}$:

(12) $$-k\frac{\partial T}{\partial n}=0\;\;\;\;\;\;\;\;\;\;\text{at}\;\text{boundaries}\;\Gamma_{2}\;\text{and}\;\Gamma_{3}$$

(3) the temperature condition at the left boundary $\Gamma_{4}$, that is assumed to be constant:

(13) $$\theta =\theta_{s}\;\;\;\;\;\;\;\;\;\;\text{at}\;\text{boundary}\;\Gamma_{4}$$

Moreover, the initial condition of the problem is given by:

(14) $$\theta \left(\text{x},t=0\right)=\theta_{0}\left(x\right)$$

The governing Equation (9), the boundary conditions (11)–(13), and the initial condition (14) consist of a complete partial differential equation system, which will be solved by the meshless method developed in the next section.

## 4. Solution Procedure

### 4.1. The Operator Splitting Method

Making use of the concept of operator splitting [20], the time-dependent governing Equation (9) can be expressed as a sum of two operators $L_{1}$ and $L_{2}$:

(15) $$\frac{\partial \theta }{\partial t}=L_{1}+L_{2}$$

where

(16) $$L_{1}=\frac{k}{\rho c}\nabla^{2}\theta$$

(17) $$L_{2}=f\left(\theta \right)$$

From Equation (15), a solution in time by a two-level time-stepping scheme is used in this work. Typically, the second-order Adams-Bashforth scheme [20]:

(18) $$\frac{\theta^{n+1/2}-\theta^{n}}{\Delta t}=\frac{3}{2}f\left(\theta^{n}\right)-\frac{1}{2}f\left(\theta^{n-1}\right)$$

and the second-order Adams-Moulton scheme [20]:

(19) $$\frac{\theta^{n+1}-\theta^{n+1/2}}{\Delta t}=\frac{1}{2}\left(\frac{k}{\rho c}\nabla^{2}\theta^{n+1}+\frac{k}{\rho c}\nabla^{2}\theta^{n}\right)$$

are, respectively, employed to model the nonlinear operator $L_{2}$ and the Laplacian operator $L_{1}$. In Equations (18) and (19), $\theta^{n-1}$, $\theta^{n}$, $\theta^{n+1}$, and $\theta^{n+1/2}$ are the temperature at the previous time step (n−1), the current time step (n), the next time step (n+1), and the half time step (n+1/2), respectively. $\Delta t=t^{n+1}-t^{n}$ is the length of the time step.

Adding Equation (19) to Equation (18) yields:

(20) $$\frac{\theta^{n+1}-\theta^{n}}{\Delta t}=\frac{3}{2}f\left(\theta^{n}\right)-\frac{1}{2}f\left(\theta^{n-1}\right)+\frac{1}{2}\left(\frac{k}{\rho c}\nabla^{2}\theta^{n+1}+\frac{k}{\rho c}\nabla^{2}\theta^{n}\right)$$

Further, replacing $\theta^{n}$ with $2\theta^{n}-\theta^{n}$ in Equation (20), we have:

(21) $$\frac{\theta^{n+1}+\theta^{n}-2\theta^{n}}{\Delta t}=\frac{3}{2}f\left(\theta^{n}\right)-\frac{1}{2}f\left(\theta^{n-1}\right)+\frac{k}{2\rho c}\nabla^{2}\left(\theta^{n+1}+\theta^{n}\right)$$

If a new variable $\theta^{*}$ defined by:

(22) $$\theta^{*}=\frac{\theta^{n+1}+\theta^{n}}{2}$$

is introduced, Equation (21) can be transformed to:

(23) $$\frac{2\theta^{*}}{\Delta t}-\frac{2\theta^{n}}{\Delta t}=\frac{3}{2}f\left(\theta^{n}\right)-\frac{1}{2}f\left(\theta^{n-1}\right)+\frac{k}{\rho c}\nabla^{2}\theta^{*}$$

that can be rearranged in the form:

(24) $$\nabla^{2}\theta^{*}-\frac{2\rho c}{k\Delta t}\theta^{*}=-\frac{\rho c}{k}\left[\frac{3}{2}f\left(\theta^{n}\right)-\frac{1}{2}f\left(\theta^{n-1}\right)\right]-\frac{2\rho c}{k\Delta t}\theta^{n}$$

Equation (24) is a type of modified Helmholtz equation and $\theta^{*}$ is a generalized function to be determined at each time step. The right nonhomogeneous term in Equation (24) is explicitly known by the previous values of $\theta^{n}$ and $\theta^{n-1}$. Subsequently, the values of $\theta^{n+1}$ can be obtained through Equation (22). Unlike the backward time-stepping scheme, this scheme needs the function values at step (*n*) and the previous step (*n*-1). Therefore, it cannot start by itself. The function value at the first time step can be evaluated by the extrapolated explicit forward Euler scheme presented below [20]

(25) $$\frac{\partial \theta }{\partial t}=\frac{\theta^{1}-\theta^{0}}{\Delta t}$$

Then we have:

(26) $$\rho c\frac{\theta^{1}-\theta^{0}}{\Delta t}=\frac{k}{\rho c}\nabla^{2}\theta^{1}+f\left(\theta^{0}\right)$$

If we set the assumed initial guess $\theta_{0}$, the value $\theta_{1}$ for the first time step can be calculated according to Equation (26). Furthermore, the time iteration can be commenced from Equation (24).

For the sake of simplicity, Equation (24) is rewritten as:

(27) $$\nabla^{2}\theta^{*}-\lambda^{2}\theta^{*}=F$$

with

(28) $$\lambda^{2}=\frac{2\rho c}{k\Delta t}$$

and

(29) $$F=-\frac{\rho c}{k}\left[\frac{3}{2}f\left(\theta^{n}\right)-\frac{1}{2}f\left(\theta^{n-1}\right)\right]-\frac{2\rho c}{k\Delta t}\theta^{n}$$

As well, the boundary conditions (11)–(13) should be modified for the time iteration so that a complete partial differential equation system can be formed with the conjunction of the governing Equation (27) and the modified boundary conditions below.

(30) $$\{\begin{array}{c} \theta^{*}=\frac{\theta_{c}+\theta^{n-1}}{2}\;\;\;\;\text{at}\;\text{boundary}\;\Gamma_{1}\\ -k\frac{\partial \theta^{*}}{\partial n}=0\;\;\;\;\text{at}\;\text{boundaries}\;\Gamma_{2}\;\text{and}\;\Gamma_{3}\\ \theta^{*}=\frac{\theta_{s}+\theta^{n-1}}{2}\;\;\;\;\text{at}\;\text{boundary}\;\Gamma_{4} \end{array}$$

### 4.2. Solution of the Modified Helmholtz System

In this subsection, the dual reciprocity method (DRM) using RBFs and the MFS using fundamental solutions are used to solve the modified Helmholtz equation system (27)–(30). Both methods are based on boundary or internal collocation and have been successfully applied to similar nonhomogeneous problems [17,28,29,30]. The solution method is described in detail below.

First, the DRM is introduced by simply setting:

(31) $$b(\text{x})=\lambda^{2}\theta^{*}(\text{x})+F$$

and then Equation (27) can be expressed as the following nonhomogeneous Laplace equation:

(32) $$\nabla^{2}\theta^{*}(\text{x})=b(\text{x})$$

According to the linear feature of the Laplacian operator, the solution to Equation (32) can be expressed as [28,29]:

(33) $$\theta^{*}(\text{x})=\theta_{h}(\text{x})+\theta_{p}(\text{x})$$

where $\theta_{h}(\text{x})$ is a homogeneous solution satisfying:

(34) $$\nabla^{2}\theta_{h}(\text{x})=0$$

and $\theta_{p}(\text{x})$ is a particular solution satisfying:

(35) $$\nabla^{2}\theta_{p}(\text{x})=b(\text{x})$$

Generally, the particular solution cannot be determined exactly. In order to find the approximated particular solution, the RBF approach is employed [31,32,33,34]. In this method, the source term b(x) is generally approximated by a series of RBFs in the domain of interest:

(36) $$b\left(\text{x}\right)=\sum_{i=1}^{M}\alpha_{i}\phi_{i}\left(r\right)$$

where $\phi_{i}$ stands for a set of radial basis functions that are defined in terms of the Euclidian distance *r* between any two interpolation points located in the domain, and $\alpha_{i}$ are the corresponding interpolating coefficients. *M* is the number of interpolation points.

Then, the particular solution of Equation (35) is represented in a form similar to that in Equation (36) [31,32,33,34]:

(37) $$\theta_{p}(\text{x})=\sum_{i=1}^{M}\alpha_{i}\Phi_{i}(r)$$

where $\Phi_{i}(r)$ are a set of particular solution kernels satisfying the following differential equation:

(38) $$\nabla^{2}\Phi_{i}(r)=\phi_{i}(r)$$

In our analysis, a one-order thin plate spline (TPS) is employed for RBF interpolation. In this case, the expressions of the basis function and the particular solution kernel can be written as [28]:

(39) $$\begin{array}{l} \phi_{i}\left(r\right)=r^{2}\ln r\\ \Phi_{i}(r)=\frac{2\ln r-1}{32}r^{4} \end{array}$$

On the other hand, the homogeneous solution satisfying Equation (34) can be obtained by means of the MFS, in which the linear combination of fundamental solutions in terms of a series of source points ${\text{s}}_{j}$ outside the domain is used to approximate the homogeneous solution at an arbitrary field point x, that is:

(40) $$\theta_{h}\left(\text{x}\right)=\sum_{j=1}^{N}\beta_{j}G_{j}(\text{x})$$

where N is the number of boundary collocation points, $\beta_{j}$ are source intensity and $G_{j}(\text{x})=G(\text{x},s_{j})$ is the fundamental solution to the linear Laplacian operator [35]:

(41) $$\nabla^{2}G\left(\text{x},{\text{s}}_{j}\right)+\delta \left(\text{x},{\text{s}}_{j}\right)=0$$

and has the form:

(42) $$G_{j}(\text{x})=-\frac{1}{2\pi }\ln\sqrt{{\left(x-x_{s}^{j}\right)}^{2}+{\left(y-y_{s}^{j}\right)}^{2}}$$

Finally, with the obtained particular and homogeneous approximations, the full solution can be written in the form:

(43) $$\theta^{*}(\text{x})=\sum_{i=1}^{M}\alpha_{i}\Phi_{i}(\text{x})+\sum_{j=1}^{N}\beta_{j}G_{j}(\text{x})$$

The normal derivative of the full solution can then be given by:

(44) $$\frac{\partial \theta^{*}\left(\text{x}\right)}{\partial n}=-\sum_{i=1}^{M}\alpha_{i}\frac{\partial \Phi_{i}(\text{x})}{\partial n}-\sum_{j=1}^{N}\beta_{j}\frac{\partial G_{j}(\text{x})}{\partial n}$$

For the purpose of simplicity, Equations (43) and (44) are written in the matrix form:

(45) $$\theta^{*}(\text{x})={\text{U}}^{*}(\text{x})\text{c}$$

(46) $$\frac{\partial \theta^{*}(\text{x})}{\partial n}={\text{Q}}^{*}(\text{x})\text{c}$$

where

(47) $${\text{U}}^{*}(\text{x})=\left[\begin{array}{llllll} \Phi_{1}(\text{x}) & \cdots & \Phi_{M}(\text{x}) & G_{1}(\text{x}) & \cdots & G_{N}(\text{x}) \end{array}\right]$$

(48) $$Q^{*}(\text{x})=\left[\begin{array}{llllll} -\frac{\partial \Phi_{1}(\text{x})}{\partial n} & \cdots & -\frac{\partial \Phi_{M}(\text{x})}{\partial n} & -\frac{\partial G_{1}(\text{x})}{\partial n} & \cdots & -\frac{\partial G_{N}(\text{x})}{\partial n} \end{array}\right]$$

(49) $${\text{c}}^{T}=\left[\begin{array}{llllll} \alpha_{1} & \cdots & \alpha_{M} & \beta_{1} & \cdots & \beta_{N} \end{array}\right]$$

Then, applying Equations (45) and (46) to the governing Equation (27) at *M* interpolation points in the domain and the boundary conditions (11)−(13) at *N* boundary collocation points leads to following system of equations:

(50) $$\{\begin{array}{l} \left[{\text{B}}^{*}({\text{x}}_{i})-\lambda^{2}{\text{U}}^{*}({\text{x}}_{i})\right]\text{c}=F({\text{x}}_{i})\;\;\;\;\;\;i=1\to M\\ {\text{U}}^{*}({\text{x}}_{j})\text{c}=\frac{\theta_{c}+\theta^{n-1}}{2}\;\;\;\;\;\;\;\;\;\;\;\;\;\;\;\;\;\;\;\;\;j=1\to N_{1}\\ {\text{Q}}^{*}({\text{x}}_{k})\text{c}=0\;\;\;\;\;\;\;\;\;\;\;\;\;\;\;\;\;\;\;\;\;\;\;\;\;\;\;\;\;\;\;\;\;\;\;\;\;\;k=1\to N_{2}\\ {\text{Q}}^{*}({\text{x}}_{l})\text{c}=0\;\;\;\;\;\;\;\;\;\;\;\;\;\;\;\;\;\;\;\;\;\;\;\;\;\;\;\;\;\;\;\;\;\;\;\;\;\;\;\;l=1\to N_{3}\\ {\text{U}}^{*}({\text{x}}_{m})\text{c}=\frac{\theta_{s}+\theta^{n-1}}{2}\;\;\;\;\;\;\;\;\;\;\;\;\;\;\;\;\;\;m=1\to N_{4} \end{array}$$

where $N_{i}\;(i=1,2,3,4)$ are respectively the number of collocation points on the four edges of the rectangular domain (see Figure 2) and $N_{1}+N_{2}+N_{3}+N_{4}=N$. ${\text{B}}^{*}$ is the Laplacian operator matrix in the form:

(51) $${\text{B}}^{*}({\text{x}}_{i})=\nabla^{2}{\text{U}}^{*}({\text{x}}_{i})=\left[\begin{array}{llllll} \phi_{1}({\text{x}}_{i}) & \cdots & \phi_{M}({\text{x}}_{i}) & 0 & \cdots & 0 \end{array}\right]$$

The unknown coefficient vector **c** can be determined from linear equation system (50), and then the temperature variable $\theta^{*}$ at each time step can be calculated from Equation (43) or (45). Due to the symmetry of the bioheat model in the rectangular domain, only half of the domain is chosen as the solution domain. Figure 2 shows an illustration of the 32 collocations, 32 source points and 63 interpolation points for the half rectangular domain in our calculation.

## 5. Conclusions

In this paper, an operator splitting technique coupled with the dual reciprocity method and the method of fundamental solutions is presented to develop a mesh-free algorithm for solving the transient nonlinear bioheat transfer in a 2D model of skin tissue with a temperature-dependent blood perfusion rate. Use of the operator splitting technique, including two-level second-order time-stepping schemes, makes it possible to establish an accurate and convergent solution procedure for transient and nonlinear cases, and then the dual reciprocity method and the method of fundamental solutions are respectively employed to solve the obtained modified Helmholtz equation system at each time step. This meshless method is dependent on the internal interpolating points and boundary collocation points of the domain only and thus is really meshless and dimension-independent. The numerical results demonstrate the accuracy and efficiency of the meshless method in the analysis of the transient nonlinear bioheat transfer problem under consideration, with very few interpolation and collocation points. Moreover, the sensitivity analysis of temperature sensitivity to the constants in the linear and exponential expressions of the blood perfusion rate demonstrates the increase in the constant $a_{2}$ in the linear case. It is found that the exponential case has a more significant influence on the tissue temperature distribution than the constant $a_{1}$, and an increase in its value results in a relatively fast increase in the tissue temperature in the region close to the outer surface and simultaneously, the peak temperature value decreases. This reflects the regulation and protection effect of the blood perfusion rate in biological tissue.
